# Efavirenz-Induced Hepatitis Mimicking Autoimmune Hepatitis: A Case Report

**DOI:** 10.7759/cureus.99540

**Published:** 2025-12-18

**Authors:** Adibul H Chowdhury, Dristy Chowdhury, Israt Jahan, Adaze Woghiren, Mohamed Saleh

**Affiliations:** 1 Internal Medicine, University Hospital of Coventry and Warwickshire NHS Foundation Trust, Coventry, GBR; 2 Medicine, Medway NHS Foundation Trust, Kent, GBR; 3 Oral and Maxillofacial Surgery, University Hospital of Coventry and Warwickshire NHS Foundation Trust, Coventry, GBR; 4 Gastroenterology, Medway NHS Foundation Trust, Kent, GBR

**Keywords:** adverse drug reaction (adr), antiretroviral therapy (art), autoimmune hepatitis, diagnostic dilemma, drug-induced liver injury (dili), efavirenz

## Abstract

Efavirenz is a widely used and effective non-nucleoside reverse transcriptase inhibitor (NNRTI) for the treatment of HIV. Hepatotoxicity, although recognized, typically occurs early in the course of NNRTI therapy. However, delayed liver injury resembling autoimmune hepatitis is rare. We report a 62-year-old HIV-positive man on long-term efavirenz who developed new, asymptomatic elevations in liver enzymes after years of stable antiretroviral therapy. Work-up revealed elevated levels antinuclear antibodies (ANA) IgG after exclusion of metabolic and viral hepatitis. Although biopsy findings suggested autoimmune hepatitis, the absence of other autoimmune features and the patient’s stable HIV control prompted reconsideration of the diagnosis.

A multidisciplinary team suspected efavirenz-related liver injury, and the drug was replaced with Raltegravir while the rest of the antiretroviral therapy (ART) regimen was maintained. Immunosuppression was withheld to observe the biochemical response. Following Efavirenz withdrawal, liver enzymes, IgG, and ANA levels normalized, and liver stiffness on FibroScan improved. This case highlights the challenge of distinguishing autoimmune hepatitis from drug-induced liver injury when autoimmune markers are present and emphasizes the importance of considering late ART toxicity, as timely drug withdrawal can lead to full recovery without immunosuppression.

## Introduction

The primary HIV infection management is antiretroviral treatment (ART). ART comprises two nucleoside reverse transcriptase inhibitors (NRTIs), tenofovir and emtricitabine, together with a non-nucleoside reverse transcriptase inhibitor (NNRTI) or an integrase strand transfer inhibitor [1]. Efavirenz is the most commonly prescribed NNRTI for the treatment of prevalent HIV strains, demonstrating high antiviral efficacy and good tolerability [2,3]. It is given at 600 mg at bedtime to limit the central nervous system side effects. Despite its high tolerability, efavirenz can damage the liver in 1-6% individuals, with severe damage in 0.5-1% [4,5]. Mitochondrial toxicity due to oxidative stress, a reactive metabolite, or an idiosyncratic reaction due to CYP2B6 polymorphisms has been proposed as a mechanism. Hepatitis toxicity generally occurs between 4 and 12 weeks of treatment. Patients may present with fatigue, nausea, periumbilical pain, elevated aspartate aminotransferase (ALT) and aspartate aminotransferase (AST), and sometimes jaundice [4,6].

Individuals who consume alcohol, are co-infected with hepatitis B or C viruses, have a slow-metabolizer CYP2B6 genotype, or are receiving other hepatotoxic medications are at increased risk of efavirenz-induced liver injury [4,7,8]. The diagnosis of drug-induced liver injury (DILI) requires exclusion of other causes, such as viral hepatitis or opportunistic infections, and may be supported by the RUCAM (Roussel Uclaf Causality Assessment Method) scale [9]. Efavirenz-related hepatotoxicity can impair adherence, necessitate ART regimen changes, and rarely progress to fulminant hepatic failure [10]. Management of such events includes early drug discontinuation, substitution with alternative agents, provision of supportive care, and cautious rechallenge when appropriate [6]. Awareness of this adverse effect, particularly in low-resource settings, facilitates safer antiretroviral therapy selection and improved clinical outcomes [10].

Although efavirenz-related hepatotoxicity is well recognized, drug-induced liver injury may occasionally mimic autoimmune hepatitis, presenting with positive autoantibodies and similar histopathological findings. This overlap can make diagnosis challenging and may lead to confusion between true autoimmune disease and medication-related toxicity. Recognizing this possibility is important when assessing new liver abnormalities in patients on long-term ART.

## Case presentation

A 62-year-old man of Zambian origin living in the United Kingdom since 1994 was found to be HIV positive while being evaluated for constitutional symptoms. The patient had a history of gout and diet-controlled type 2 diabetes mellitus. Following diagnosis, he was initiated on efavirenz, emtricitabine, and tenofovir disoproxil fumarate (Truvada), a regimen that was well tolerated during the initial years of therapy.

During routine screening in August 2018, the patient’s interferon-gamma release assay (IGRA, T-SPOT.TB test) was positive for latent tuberculosis. The preliminary liver function tests, full blood count, and renal function were all normal at the start of the study. He completed a six-month course of isoniazid 300mg with pyridoxine 10mg without any side effects. In March 2019, imaging of the chest was performed, which was normal. Repeat liver function tests (LFT) were stable. Ultimately, the patient completed tuberculosis follow-up and was discharged from care.

In May 2022, the patient was found to have significantly elevated liver enzymes during routine HIV care, despite being asymptomatic. Tests showed ALT and AST levels of 167 IU/L (normal range: 0-40 IU/L) and 154 IU/L (normal range: 0-40 IU/L), respectively. Gamma-glutamyl transferase (GGT) was at 147 IU/L (normal range <60 IU/L). Also, ALP was at 178 IU/L (normal range: 30-130 IU/L). Bilirubin, albumin, and INR were normal (Table 1). Blood counts and renal parameters remained unremarkable. The abdominal ultrasound report showed that the liver echotexture and size were normal. The patient reported intermittent use of naproxen for gout. He had no history of alcohol use or exposure to other hepatotoxins.

**Table 1:** Liver function and key laboratory parameters (2018–2025) showing marked elevation of ALT, AST, and GGT in 2022, with complete normalization after efavirenz withdrawal. ALT: Alanine transaminase; AST: Aspartate transaminase; GGT: Gamma-glutamyl transferase; ALP: Alkaline phosphatase; INR: International normalized ratio

Date	ALT ( 0-40 IU/L)	AST ( 0-40 IU/L)	GGT ( <60 IU/L)	ALP ( 30-130 IU/L)	Total Bilirubin ( <21 µmol/L)	Albumin ( 30-50 g/L)	Prothrombin Time ( 9.5-13 sec)	INR ( 0.8-1.2 )
Aug 2018	22	24	22	66	8	42	12.8	1
May 2022	167	154	147	178	8	43	13.7	1.1
Jun 2023	18	29	28	72	11	44	12.2	1
Mar 2024	14	22	30	80	11	-	-	-
Jan 2025	19	21	48	78	11	-	-	-

Further workup in June 2022 revealed a strongly positive antinuclear antibody (ANA) with a titer of 1:320 and a homogeneous pattern; serum IgG was elevated at 19.1 g/L (normal range: 6-16 g/L). The results of other autoimmune tests were negative. These tests include anti-DsDNA, anti-LKM, anti-smooth muscle, and antimitochondrial antibodies. Test results for serum ceruloplasmin, alpha-1 antitrypsin, and hepatitis B and C serologies were normal. Transient elastography (FibroScan) showed a liver stiffness of 6.8 kPa (F0-F1 fibrosis) and a controlled attenuation parameter (CAP) of 187 dB/m (Normal level of CAP score <238 dB/m) (Table 2). A liver biopsy performed at that time demonstrated a moderate chronic portal inflammatory infiltrate composed mainly of lymphocytes and plasma cells with a few eosinophils. There was continuous interface hepatitis and scattered lobular inflammation without confluent necrosis. Minimal steatosis was present, along with a mild ductular reaction. Fibrosis was mild (stage 2/6 on the modified Ishak scale) [11]. Overall, the histology was reported as compatible with autoimmune hepatitis.

**Table 2:** Immunological and FibroScan results improved progressively following modification of ART. Normal level of liver stiffness <5.5 kPa ( < 5.5 kPa → F0 (no fibrosis), 5.5–7.0 kPa: Borderline/mild fibrosis (F0–F1), ≥ 7.0 kPa → Increasing likelihood of significant fibrosis.

Date	IgG (g/L)	ANA	Liver Stiffness (kPa)	CAP Score (dB/m)	Fibrosis Stage
Jun 2022	19.1	Positive (1:320, homogeneous)	6.8	187	F0–F1 (mild fibrosis)
Jun 2023	15.8	Negative	5.7	288	F0 (no significant fibrosis)
Jan 2025	16.2	Negative	4.3	221	F0 (resolved fibrosis)

The initial working diagnosis was autoimmune hepatitis, and management was planned accordingly; however, given the temporal association between prolonged efavirenz exposure and the abrupt elevation in liver enzymes, drug-induced liver injury was also considered in the differential diagnosis. A RUCAM (Roussel Uclaf Causality Assessment Method) evaluation was performed, yielding a score of 7, which placed the liver injury in the ‘probable’ category for drug-induced hepatotoxicity. Following multidisciplinary discussions with the hepatology and HIV teams, efavirenz was replaced with raltegravir, while emtricitabine and tenofovir were continued. Immunosuppressive therapy was deferred to assess the response to antiretroviral therapy modification.

The patient was advised on lifestyle modification and adopted dietary changes for weight and glycemic control, as well as limiting alcohol consumption to fewer than 10 units per week. The hepatology care plan has included these interventions as reinforcers.

In June 2023, follow-up testing showed complete normalization of liver enzymes with improved immunological markers (Tables 1 and 2). The levels of IgG antibodies fell to 15.8 g/L, and the ANA test was negative. When the FibroScan was done, it showed a liver stiffness of 5.7 kPa and CAP of 288 dB/m (F0), consistent with mild steatosis without fibrosis progression (Table 2).

At follow-up in March 2024 and January 2025, the liver biochemistry normalized, and the IgG levels were stable. Moreover, the patient’s liver stiffness (4.3 kPa) and the CAP score (221 dB/m) improved, indicating resolution of the fibrosis and improvement of the hepatic steatosis. The patient remains clinically well and has no recurrence of liver disease. He has sustained viral suppression of HIV on raltegravir and emtricitabine/tenofovir (Truvada) replacement therapy. He still attends annual follow-up appointments in the hepatology and infectious diseases clinic without any complications.

## Discussion

Efavirenz, by virtue of its potent efficacy, favorable resistance profile and safety, and cost-effectiveness, is still a key component of antiretroviral therapy (ART) in many of the low- and middle-income countries. However, mounting clinical evidence, including the longitudinal cohort study by Peluso et al. [10], has shown that liver function test (LFT) abnormalities are a significant side effect of efavirenz, especially with prolonged usage.

After years of stable therapy, the patient developed marked transaminase elevations. These findings posed a diagnostic dilemma, given a strongly positive ANA, elevated IgG, and other work-up findings consistent with autoimmune hepatitis. The first examination of the liver showed signs resembling autoimmune hepatitis, which could wrongly direct doctors towards a treatment that suppresses the immune system.

Yet the lack of other autoimmune markers and symptoms, along with the fact that the patient’s HIV is stable, broadened the differential. Based on a multidisciplinary review of the case and the strong biochemical and immunological response upon the withdrawal of efavirenz, drug-induced liver injury was suspected. The timing of the damage, which occurs years after the initiation, agrees with the findings of Peluso et al. [10] who showed that LFT abnormalities are sustained or delayed in those treated from early HIV infection.

This case underscores several diagnostic principles. Medical professionals must remember that drug-induced liver disease could even occur in long-standing ART when autoimmune markers are ambiguous. There is an important and clinically relevant overlap between autoimmune hepatitis and DILI. This is especially relevant in ART-related hepatotoxicity, where liver biopsy may not be conclusive. Published literature on autoimmune hepatitis reports typical histological features, including interface hepatitis, lymphoplasmacytic infiltrates, and early fibrosis, that were also present in our patient’s biopsy. This overlap highlights why distinguishing autoimmune hepatitis from drug-induced liver injury can be challenging in cases like this. In conclusion, the RUCAM is a useful tool in the structured assessment of DILI and should be used early in complex hepatology cases [9].

The patient’s favorable outcome following the substitution of efavirenz with raltegravir, marked by normalization of liver enzymes and improved FibroScan scores, highlights the reversibility of ART-induced hepatotoxicity when promptly recognized and addressed. The patient’s case also highlights the need for continuous monitoring and close collaboration between HIV and hepatology services to prevent long-term treatment-related complications. Critically, this scenario presents a clinical dilemma: in cases of drug-induced liver injury mimicking autoimmune hepatitis (AIH), should clinicians initiate immunosuppressive therapy first, or prioritize drug withdrawal, even when autoimmune markers and histology suggest AIH? In this case, we decided to stop efavirenz instead of starting immunosuppression. In the end, this led to complete recovery and saved unnecessary treatment. This result, though, brings to light the diagnostic ambiguities that often accompany such overlapping presentations, as well as the need for clearer clinical guidance to enable more nuanced, individual decision-making in similar cases.

## Conclusions

This case shows how efavirenz can cause liver injury that looks very similar to autoimmune hepatitis, making the diagnosis challenging. The overlapping laboratory and biopsy findings could have led to misdiagnosis; however, the patient’s full recovery following discontinuation of efavirenz confirmed the true etiology. His liver tests, immunological markers, and FibroScan results all returned to normal once the medication was withdrawn, confirming a drug-induced process rather than genuine autoimmune disease. This case underscores the importance of considering antiretroviral-related hepatotoxicity when new liver abnormalities arise, even after years of stable therapy. It also emphasizes the value of close collaboration between HIV and hepatology specialists to ensure timely recognition and avoid unnecessary treatment.
